# Association Between Declined Offers of Deceased Donor Kidney Allograft and Outcomes in Kidney Transplant Candidates

**DOI:** 10.1001/jamanetworkopen.2019.10312

**Published:** 2019-08-30

**Authors:** S. Ali Husain, Kristen L. King, Stephen Pastan, Rachel E. Pazter, David J. Cohen, Jai Radhakrishnan, Sumit Mohan

**Affiliations:** 1 Division of Nephrology, Department of Medicine, Columbia University Medical Center, New York, New York; 2 The Columbia University Renal Epidemiology (CURE) Group, New York, New York; 3 Renal Division, Department of Medicine, Emory University School of Medicine, Atlanta, Georgia; 4 Division of Transplantation, Department of Surgery, Emory University School of Medicine, Atlanta, Georgia; 5 Department of Epidemiology, Rollins School of Public Health, Emory University, Atlanta, Georgia; 6 Department of Epidemiology, Columbia University Mailman School of Public Health, New York, New York

## Abstract

**Importance:**

In the United States, substantial disparities in access to kidney transplant exist for wait-listed candidates with end-stage renal disease. The implications of transplant centers’ willingness to accept kidney offers for access to transplant and mortality outcomes are unknown.

**Objective:**

To determine the outcomes for wait-listed kidney transplant candidates after the transplant center’s refusal of a deceased donor kidney offer.

**Design, Setting, and Participants:**

This cohort study obtained data from the United Network for Organ Sharing Potential Transplant Recipient data set on all deceased donor kidney offers in the United States made between January 1, 2008, and December 31, 2015. The final study cohort included adult patients who were wait-listed for kidney transplant and received at least 1 allograft offer during the study period (N = 280 041). Data analysis was conducted from June 1, 2018, to March 30, 2019.

**Exposure:**

Candidate state of residence.

**Main Outcomes and Measures:**

Waiting list outcome event groups included received deceased donor allograft, received living donor allograft, died while on the waiting list, removed from the waiting list without a transplant, or still on the waiting list at the end of follow-up.

**Results:**

Among the 280 041 kidney transplant candidates included in the study, the mean (SD) age at wait-listing was 51.1 (13.1) years, and male patients were predominant (171 517 [61.2%]). In this cohort, 81 750 candidates (29.2%) received a deceased donor kidney allograft, 30 870 (11.0%) received a living donor allograft, 25 967 (9.3%) died while on the waiting list, and 59 359 (21.2%) were removed from the waiting list. Overall, 10 candidates with at least 1 previous allograft offer died each day during the study period. Time to first offer was similar for candidates who received deceased donor kidney allograft compared with those who died while waiting (median [interquartile range {IQR}] time, 79 [16-426] days vs 78 [17-401] days, respectively). Deceased donor allograft recipients had a median of 17 offers (IQR, 6-44) over 422 days (IQR, 106-909 days), whereas candidates who died while waiting received a median of 16 offers (IQR, 6-41) over 651 days (IQR, 304-1117 days). Most kidneys (84%) were declined on behalf of at least 1 candidate before being accepted for transplant. As reported by centers, organ or donor quality concerns accounted for 8 416 474 (92.6%) of all declined offers, whereas offers were infrequently refused because of patient-related factors (232 193 [2.6%]), logistical limitations (49 492 [0.5%]), or other concerns. The odds of death after an offer and the median number of offers received prior to death varied considerably by state.

**Conclusions and Relevance:**

This study found that transplant candidates appeared to receive a large number of viable deceased donor kidney offers that were refused on their behalf by transplant centers, potentially exacerbating the detrimental consequences of the organ shortage; increased transparency in organ allocation process and decisions may improve patient-centered care and access to kidney transplant.

## Key Points

**Question:**

What are the outcomes for wait-listed kidney transplant candidates after a transplant center’s refusal to accept a deceased donor kidney offer on their behalf?

**Findings:**

In this cohort study of 280 041 wait-listed kidney transplant candidates who received at least 1 deceased donor kidney offer, approximately 30% of these candidates eventually died or were removed from the waiting list before receiving an allograft. Deceased donor kidney allograft recipients received a median of 17 offers over 422 days, whereas candidates who died while waiting received a median of 16 offers over 651 days, and the odds of death on the waiting list after receiving an offer varied across the United States.

**Meaning:**

This study suggests that a large number of deceased donor kidney offers are received by candidates but are declined on their behalf, resulting in what appears to be many missed opportunities for a transplant before death or removal from the waiting list.

## Teaser

This cohort study examines outcomes for wait-listed kidney transplant candidates after a transplant center’s refusal of a deceased donor kidney offer.

## Introduction

The widespread geographic variation in the incidence of end-stage renal disease (ESRD) and access to kidney transplant in the United States is well documented.^1,2,3^ Although geographic disparities in kidney transplant are believed to be largely associated with regional variations in organ availability and ESRD prevalence, to date, the contribution of the differences in acceptance rates of deceased donor kidney offers to these disparitites is unknown.^1,2,4^

At present, organ offers to transplant candidates are made to the transplant center at which a given candidate is wait-listed. A center has the ability to decline the offer on the candidate’s behalf without informing the candidate of the offer or the reason it was declined. Despite the advantages of earlier transplant for patients with ESRD, deceased donor kidneys are offered to a median of 7 different candidates before being accepted for transplant, with one-quarter of transplanted deceased donor kidneys offered first to at least 73 candidates.^5,6,7,8,9^ Organ offers are often declined on the basis of center-level organ selection practices rather than a detailed assessment of the advantages to each individual candidate of receiving that kidney allograft, including a shorter time receiving dialysis.

To date, the implication of transplant centers’ ability to decline such offers for candidates’ access to transplant is not known, and the consequences for candidates who remain wait-listed when the organs are refused on their behalf have not been studied, to our knowledge. We examined the outcomes for actively wait-listed patients who received at least 1 offer for a deceased donor kidney that was eventually transplanted in another patient with a lower priority on the computer-generated rank-order list of matched candidates for that organ (match run).

## Methods

### Study Design and Participants

This study was approved by the institutional review board of Columbia University Medical Center, New York. Given that the analysis was performed using deidentified data from a national registry of wait-listed patients and allograft recipients, informed consent could not be obtained.

We obtained data from the United Network for Organ Sharing (UNOS) Potential Transplant Recipient data set. This UNOS data set includes an ordered list of all matched kidney transplant candidates to whom deceased donor kidneys were offered, including the offers ultimately accepted for transplant. Match-run data for discarded deceased donor kidneys are excluded from the Potential Transplant Recipient data set, and candidates with a lower priority on the waiting list than the patient who received the kidney are excluded from the match run data (see eAppendix in the Supplement). Offers are given to only wait-listed patients with an active status.^10^ We excluded matches that were automatically declined by the allocation system bypasses (eg, directed donation, payback agreements, and military allocation) because the candidates were unable to receive these deceased donor kidneys although they were recorded as offers (eFigure 1 in the Supplement). We used the UNOS Standard Transplant Analysis and Research file for demographic and outcomes data on candidates and donors and followed the Strengthening the Reporting of Observational Studies in Epidemiology (STROBE) reporting guidelines.

The study cohort included all adult candidates in the United States who received 1 or more deceased donor kidney offers from January 1, 2008, to December 31, 2015. We excluded offers received after the candidate’s documented death date, transplant date, or waiting list removal date.^11^ We also excluded candidates whose state of residence at listing was missing or outside of the 50 US states and Washington, DC, because state was our primary exposure of interest.

We classified the final cohort of candidates into 5 event groups occurring from January 1, 2008, to December 31, 2015, as follows: received a deceased donor allograft, received a living donor allograft, died without undergoing a transplant, removed from the waiting list for a reason other than death or transplant, or still awaiting a transplant. We identified the date of each candidate’s first offer and calculated the total number of offers they received during the study window before their event date. The end of follow-up for each candidate was either transplant, death, removal from the waiting list, or end of the study (December 31, 2015).

The Estimated Post Transplant Survival (EPTS) score was calculated for each candidate at listing and first offer using the Organ Procurement and Transplantation guidelines and 2015 mapping tables.^12^ Offer refusal codes provided by transplant centers were grouped into 1 of 4 reasons for declining the offers: patient related, organ or donor quality, logistical, and immunologic or other (eTable 1 in the Supplement). Additional variable definitions are detailed in the eAppendix in the Supplement.

### Statistical Analysis

Candidate characteristics were compared across the 5 groups using analysis of variance for normally distributed continuous variables, Kruskal-Wallis tests for nonnormally distributed continuous variables, and Pearson χ^2^ tests for categorical variables. Pairwise comparisons between the death event group and each of the other 4 event groups were performed using the Bonferroni multiple comparisons adjustment as well as the Dunn test for continuous variables and multinomial regression for categorical variables. Means and SDs or medians with interquartile ranges (IQRs) are presented for continuous characteristics. Percentages are given for categorical characteristics.

Odds ratios (ORs) of dying on the waiting list by state were estimated with logistic regression, with the reference state as Maine (the state with the lowest proportion of waiting list deaths). Multivariable logistic regression, controlling for potential confounders that showed evidence of an association at the univariate level, was performed. In the final adjusted model, we calculated the OR of waiting list death by candidate state of residence after adjusting for candidate race and ethnicity, obesity category (body mass index of ≥30 or <30 [calculated as weight in kilograms divided by height in meters squared]), age, presence of diabetes and vascular disease, calculated panel reactive antibody (>80% or ≤80%), preemptive status (never received dialysis, started dialysis after preemptive listing, or receiving dialysis at listing), and time (number of months from listing) to first offer.

A 2-sided α = .05 was used to assess statistical significance. All analyses were completed using Stata, version 15.1 (StataCorp LLC). Maps were created using ArcGIS ArcMap, version 10.6 (ESRI Inc). Data analysis was conducted from June 1, 2018, to March 30, 2019.

## Results

Of the 367 405 candidates on the waiting list between January 1, 2008, and December 31, 2015, a total of 280 041 eligible candidates received 1 or more offers for a deceased donor kidney during the study period (eFigure 1 in the Supplement). Among the included candidates, the mean (SD) age at listing was 51.1 (13.1) years, and male patients were predominant (171 517 [61.2%]). Median (IQR) time receiving dialysis at listing was 1.2 (0.6-2.6) years, 116 712 candidates (41.7%) had diabetes, 37 629 (13.4%) had panel reactive antibody greater than 80%, and 16 778 (6.0%) had vascular disease. Among these candidates, 81 750 (29.2%) received a deceased donor kidney allograft, 30 870 (11.0%) received a living donor allograft, 25 967 (9.3%) died while on the waiting list, and 59 359 (21.2%) were removed from the waiting list (Table 1). The remaining candidates were still wait-listed as of December 31, 2015. Median (IQR) follow-up was 755 (328-1340) days since listing.

**Table 1.** Characteristics of Transplant Candidates Who Received at Least 1 Deceased Donor Kidney Offer, 2008-2015

Variable^a^	Total	Event Group				
		Died While on Waiting List	Received Allograft From DD	Received Allograft From LD	Removed From Waiting List	Remaining on Waiting List
No. (%)	280 041	25 967 (9.3)	81 750 (29.2)	30 870 (11.0)	59 359 (21.2)	82 095 (29.3)
Age at listing, mean (SD), y	51.1 (13.1)	54.9 (11.5)	50.8 (13.1)	47.6 (13.8)	51.8 (13.5)	51.1 (12.6)
Age at first offer, mean (SD), y	51.8 (13.0)	55.8 (11.3)	51.7 (12.9)	47.9 (13.8)	52.6 (13.4)	51.5 (12.5)
Female, No. (%)	108 524 (38.8)	9746 (37.5)	32 367 (39.6)	11 552 (37.4)^b^	23 308 (39.3)	31 551 (38.4)
Ethnicity, No. (%)						
Hispanic	46 433 (16.6)	4397 (16.9)	12 221 (14.9)	4486 (14.5)	9373 (15.8)	15 956 (19.4)
Non-Hispanic	233 608 (83.4)	21 570 (83.1)	69 529 (85.1)	26 384 (85.5)	49 986 (84.2)	66 139 (80.6)
Race, No. (%)						
White	124 327 (44.4)	11 551 (44.5)	35 612 (43.6)	20 086 (65.1)	27 135 (45.7)	29 943(36.5)
Black	85 052 (30.4)	7905 (30.4)	27 063 (33.1)	4377 (14.2)	17 830 (30.0)	27 877 (34.0)
Hispanic	45 990 (16.4)	4352 (16.8)	12 079 (14.8)	4437 (14.4)	9304 (15.7)	15 818 (19.3)
Asian	19 256 (6.9)	1633 (6.3)	5373 (6.6)^b^	1524 (4.9)	4002 (6.7)^b^	6724 (8.2)
Other	5416 (1.9)	526 (2.0)	1623 (2.0)^b^	446 (1.4)	1088 (1.8)^b^	1733 (2.1)
BMI, mean (SD)	28.4 (5.5)	28.4 (5.7)	28.3 (5.5)	28.0 (5.5)	28.2 (5.5)	28.9 (5.5)
History of diabetes, No. (%)	116 712 (41.7)	15 426 (59.4)	29 443 (36.0)	8482 (27.5)	27 337 (46.1)	36 024 (43.9)
History of vascular disease, No. (%)	16 778 (6.0)	2077 (8.0)	5607 (6.9)	1503 (4.9)	3056 (5.2)	4535 (5.5)
PRA, mean (SD), %^c^	20.8 (34.4)	22.3 (35.9)	21.7 (34.9)^b^	10.3 (23.7)	21.5 (35.2)^b^	22.8 (35.6)
PRA >80%, No. (%)^c^	37 629 (13.4)	3915 (15.1)	12 086 (14.8)^b^	1215 (3.9)	8394 (14.1)	12 019 (14.6)^b^
EPTS score at listing, median (IQR)	35 (15-59)	52 (31-71)	34 (14-58)	21 (7-42)	39 (18-63)	36 (16-58)
EPTS score at first offer, median (IQR)	38 (17-63)	56 (34-76)	38 (17-63)	22 (8-44)	42 (20-68)	38 (17-61)
Dialysis vintage at listing, median (IQR), y	1.2 (0.6-2.6)	1.3 (0.7-2.7)	1.5 (0.7-3.3)	0.7 (0.4-1.5)	1.3 (0.6-2.6)	1.2 (0.6-2.3)
Dialysis vintage at first offer, median (IQR), y	1.8 (0.9-3.6)	2.1 (1.1-3.9)	2.5 (1.2-4.6)	1.0 (0.5-2.0)	1.9 (1.0-3.7)	1.4 (0.8-2.7)
Wait-listed preemptively, No. (%)	79 245 (28.3)	4767 (18.4)	19 761 (24.2)	15 178 (49.2)	15 130 (25.5)	24 409 (29.7)
Started dialysis between wait-listing and event, No. (%)^d^	14 953 (5.3)	750 (2.9)	2059 (2.5)^b^	950 (3.1)	2190 (3.7)	9004 (11.0)
Days between listing and first offer, median (IQR)	48 (13-232)	78 (17-401)	79 (16-426)^b^	34 (11-103)	62 (16-302)	30 (9-104)
Days between first offer and event, median (IQR)^d^	526 (193-1041)	651 (304-1117)	422 (106-909)	188 (83-403)	690 (326-1192)	650 (276-1255)
No. of offers before event, median (IQR)^d^	16 (5-40)	16 (6-41)	17 (6-44)	7 (3-16)	15 (6-37)	21 (8-51)
Days between first and last offers, median (IQR)	386 (122-829)	390 (140-764)	420 (103-907)	144 (40-350)	392 (149-775)^b^	490 (191-984)

Abbreviations: BMI, body mass index (calculated as weight in kilograms divided by height in meters squared); EPTS, Estimated Post Transplant Survival; DD, deceased donor; IQR, interquartile range; LD, living donor; PRA, panel reactive antibody.

^a^ All variables statistically significant for overall group differences, with *P* < .001.

^b^ Indicates a nonsignificant (*P* > .05) pairwise comparison between death group and that event group.

^c^ Calculated PRA is the candidate’s most recent calculated PRA.

^d^ *Event* is defined as death, transplant, removal from waiting list, or end of follow-up period (December 31, 2015) for those remaining on the waiting list.

A mean of 10 candidates who previously received an offer died every day during the study period. Candidates who died while on the waiting list, compared with those in other groups, were statistically significantly older, more likely to have diabetes or vascular disease, and less likely to be wait-listed before dialysis initiation (Table 1). Candidates received their first offer of an organ a median (IQR) of 48 (13-232) days after wait-listing. Median (IQR) time to first offer was similar between candidates who received a deceased donor kidney allograft (79 [16-426] days) and those who died waiting (78 [17-401] days). Candidates who underwent a deceased donor kidney transplant received a median of 17 offers (IQR, 6-44) over a median of 422 days (14 months; IQR, 106-909 days) before the transplant. Similarly, candidates who died on the waiting list received a median of 16 offers (IQR, 6-41) over a median of 651 days (21 months; IQR, 304-1117 days) before death, whereas those who were removed from the waiting list received a median of 15 offers (IQR, 6-37) while wait-listed. Candidates who eventually underwent a kidney transplant from a living donor received a median (IQR) of 7 (3-16) offers while wait-listed, with a median (IQR) of 188 (83-403) days between first offer and transplant.

Most deceased donor kidneys (84%) were declined on behalf of 1 or more candidates before being accepted for transplant, with 27% of transplanted kidneys refused for all candidates in their procuring donation service area (DSA), resulting in nonlocal use. Although 0 HLA mismatch offers represented only 49 791 offers (0.5%), 42 054 (84.5%) were declined. As reported by centers, organ or donor quality concerns accounted for 8 416 474 (92.6%) of all declined offers, whereas offers were infrequently refused because of patient-related factors (232 193 [2.6%]), logistical limitations (49 492 [0.5%]), or other concerns, a trend that was stable across the study period (eTable 2 in the Supplement). Organ or donor quality concerns remained the primary reason for declined offers across all Kidney Donor Profile Index deciles (Table 2). When comparing time to first offer between years in the study period, we found a decrease over time, with the largest change happening in 2015 (Table 3). The proportion of kidneys that were not declined on behalf of any candidate decreased in 2015.

**Table 2.** Reasons for Declining Deceased Donor Kidney Offers

KDPI Decile^a^	Reasons for Declined Offers, No. (%)^b^			
	Patient Related	Organ or Donor Quality	Logistical	Immunologic or Other
All	232 193 (2.6)	8 416 474 (92.6)	49 492 (0.5)	388 920 (4.3)
0-10	11 047 (4.2)	229 853 (87.1)	1533 (0.6)	21 383 (8.1)
11-20	20 011 (4.5)	394 128 (87.9)	2718 (0.6)	31 300 (7.0)
21-30	16 232 (2.8)	522 603 (90.7)	2945 (0.5)	34 531 (6.0)
31-40	21 722 (3.7)	540 643 (91.6)	2844 (0.5)	25 340 (4.3)
41-50	20 244 (2.6)	726 831 (92.9)	5408 (0.7)	30 045 (3.8)
51-60	23 576 (2.4)	917 430 (92.0)	7213 (0.7)	48 827 (4.9)
61-70	29 091 (2.4)	1 118 833 (93.5)	5124 (0.4)	43 836 (3.7)
71-80	28 914 (2.0)	1 337 402 (93.3)	8687 (0.6)	57 896 (4.0)
81-90	33 078 (2.3)	1 379 845 (93.8)	7396 (0.5)	51 413 (3.5)
91-100	26 841 (2.1)	1 194 896 (94.3)	5042 (0.4)	40 826 (3.2)
Unknown	1437 (2.4)	54 010 (90.7)	582 (1.0)	3523 (5.9)

Abbreviation: KDPI, Kidney Donor Profile Index.

^a^ KDPI is a relative measure of donor quality, with lower KDPI considered higher donor quality.

^b^ *P* < .001, obtained using χ^2^ test.

**Table 3.** Secular Trends in Time to First Offer and Likelihood of Kidney Refusal

Year	Time to First Offer, Median (IQR), d^a^	Kidneys Accepted With No Declines, No. (%)^b^
2008	38 (12-104)	1415 (14.0)
2009	33 (11-105)	1428 (14.4)
2010	34 (11-109)	1761 (17.4)
2011	30 (10-94)	1712 (16.3)
2012	33 (11-106)	1895 (18.4)
2013	31 (10-99)	1808 (17.0)
2014	25 (8-75)	1721 (15.8)
2015	14 (5-35)	1350 (11.7)
All	29 (9-88)	13 090 (15.6)

Abbreviation: IQR, interquartile range.

^a^ Among newly wait-listed candidates that year; *P* < .001 for comparison, obtained using Kruskal-Wallis test.

^b^ *P* < .001 for comparison, obtained using χ^2^ test.

**Figure 1.**
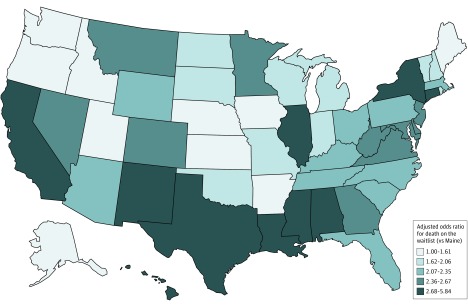
Adjusted Odds Ratio for Death While on the Waiting List After Receipt of at Least 1 Deceased Donor Kidney Offer, by Candidate State of Residence, 2008-2015 Maine, the state with the lowest odds of death while on the waiting list after receiving an offer, was the reference state. The model was adjusted for age, body mass index category, presence of diabetes, and presence of vascular disease at listing, and was adjusted for race/ethnicity, preemptive status, peak calculated panel reactive antibody, and number of days between listing and first offer.

**Figure 2.**
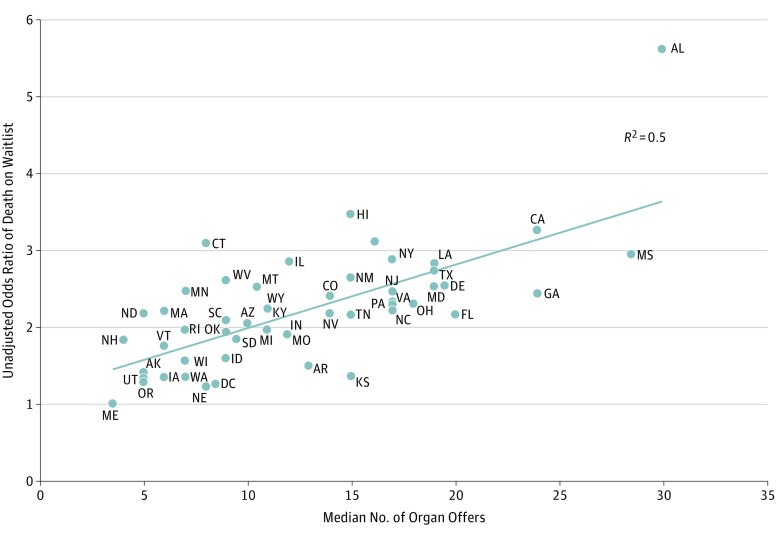
Median Number of Deceased Donor Kidney Offers to Those Who Died While on the Waiting List by Adjusted Odds of Death on the Waiting List, by Candidate State of Residence, 2008-2015. Maine, the state with the lowest odds of death on the waiting list after receiving an offer, was the reference state.

Marked state-level variability was observed in the interval between first offer and death or transplant (eFigure 2 in the Supplement) and in the likelihood of dying while having remained on the waiting list after receiving an offer. There was a statistically significant geographic heterogeneity in the OR of death while still remaining on the waiting list after receiving at least 1 offer (eFigure 3 in the Supplement). These differences persisted after adjusting for candidate demographics and comorbidities (age, race, body mass index, diabetes, vascular disease, preemptive status, and calculated panel reactive antibody) and time between wait-listing and first offer (Figure 1). In this adjusted analysis, candidates in 39 states were statistically significantly more likely to die after receiving deceased donor kidney offers than those in Maine (eTable 3 in the Supplement). The median (IQR) number of offers received before death while having remained on the waiting list also varied widely by state, ranging from 3.5 (1-11) to 30 (11-74) offers (eTable 4 in the Supplement) and correlated with the adjusted odds of death on the waiting list after receiving an offer (*r*^2^ = 0.50; *P* < .001) (Figure 2).

## Discussion

Kidney transplant provides survival advantages over dialysis for patients with ESRD, but it is limited by a shortage of organs.^13,14^ Deaths while awaiting transplant and waiting list removal before transplant are considered unfortunate consequences of the organ scarcity. Results of this study suggest that candidate deaths and waiting list removals are the consequence of not merely never having an opportunity to receive an allograft but also of refusing multiple offers to receive a donor organ that ultimately went to someone with a lower waiting list priority. Our estimate of the viable offers that candidates received is conservative, given the exclusion of all offers for all discarded deceased donor kidneys, many of which were transplantable.^15,16,17,18^

Concerns about organ or donor quality were the predominant reason given by centers for refusing deceased donor offers, even for high-quality kidneys (ie, those with low Kidney Donor Profile Index scores). Given that all included kidneys were eventually transplanted, these offer declines suggest that centers have varying thresholds for organ acceptability, which in turn has implications for patients wait-listed at centers that are more conservative in their organ quality assessments. The number of times a kidney is declined has been shown to not be associated with posttransplant outcomes, calling into question the validity of subjective decisions to refuse offers.^19^

The short median time to first offer for each group is noteworthy given the long wait times for deceased donor kidney allograft. This time is likely associated with the 27% of kidneys that were transplanted outside of their procuring DSA. Offers for these kidneys must have first exhausted the list of eligible candidates within the DSA, including candidates with short waiting times. Candidates would likely be surprised to learn that any kidney they could have received, regardless of quality, was instead refused on their behalf and allowed to be sent elsewhere in the United States for transplant. The time to first organ offer also varied across the country, consistent with the known geographic variation in access to deceased donor kidney allograft.^1,2,3^ A transplant center’s decision to decline a deceased donor kidney offer may be informed in part by the perception of the severity of the organ shortage (ie, the perceived probability of receiving a superior offer in the near future) in their region. Furthermore, centers often make broad decisions about an organ’s suitability for transplant rather than consider the advantages for each candidate on their waiting list. This perspective tends to lead to the export of kidneys from a DSA or region despite the potential advantages for patients with low waiting list priority and long expected wait time.

We found an association between the number of offers declined per candidate and the odds of death at the state level. Patients presumably spent more time on the waiting list accruing offers, and thus this association between increased number of declined offers and death is not surprising. This finding, however, suggests that centers expected these patients to receive better organ offers in the near future, justifying the detrimental implications of continued dialysis exposure.^20^ However, because many candidates with declined offers eventually died or were removed from the waiting list, it is difficult to argue that they would not have been better served by acceptance of any of their earlier offers. Because of the adverse effect of longer pretransplant dialysis exposure on posttransplant outcomes, even candidates who eventually received allografts would likely have experienced better expected survival and quality of life from accepting an earlier offer.^8,20,21^ Although organ or donor quality concerns were the primary reason for refusing offers, these missed opportunities for an earlier transplant occur despite evidence that receiving even marginal-quality kidneys provides survival and quality-of-life advantages over ongoing wait-listing for most candidates.^13,22,23,24,25^ Making the adverse consequences of declining offers more evident to all parties is likely necessary in order to change clinician and patient behavior.

Ideal management of a transplant center’s waiting list should involve the deactivation, even briefly, of candidates who are not ready or able to receive an allograft, to prevent them from receiving organ offers until reactivation. Nevertheless, centers could decline offers for such individuals if they remain active, and, in these circumstances, declining the offer is appropriate. However, only a small minority (<3%) of declined offers were attributed to patient status.

These findings are most concerning for the wait-listed individuals. Because the primary concern of wait-listed candidates is the time it takes to get a kidney allograft, candidates would likely be perplexed to discover that any deceased donor kidney was declined on their behalf.^26^ Patient-centered decision-making processes should prioritize the survival and quality-of-life advantages associated with transplants and should favor an early transplant.^14^ In contrast, the current regulatory framework for a transplant in the United States is focused on short-term patient and graft survival for the subset of patients with ESRD who are fortunate enough to be wait-listed and subsequently receive an allograft. This focus may be associated with the risk aversion at many centers and their reluctance to use anything but ideal deceased donor kidneys to ensure excellent short-term outcomes, although this practice is inconsistent with optimizing overall candidate survival or with patient preferences for minimizing wait time.^26^ Centers are becoming more conservative in their organ acceptance, as reflected in the decreased proportion of kidneys accepted by the highest-priority candidate on the match run and the increased proportion of kidneys discarded over time.^15,27^ The implementation of the new kidney allocation system^10^ in December 2014 seems to have been followed by a substantial change in organ acceptance, although more data are needed to determine if this change is transient.

Several potential policy changes could create a patient-centered organ offer process, disincentivize offer declines, and potentially decrease organ discards. First, individual candidates should be made aware of all offers declined on their behalf. Although the time constraints of organ allocation are not conducive to real-time shared decision-making, alternative strategies merit consideration. Routine post hoc reporting to patients and their nephrologists about declined offers might improve communication and patient engagement while prompting centers to reconsider how or when to decline offers. These reports could include the reasons the center provided for refusing the offer. Second, center-level data regarding offer-acceptance trends and minimum acceptance criteria (which account for many declined offers^9^) could be made publicly available along with currently reported transplant center metrics. Such a policy may allow candidates to identify centers whose offer-acceptance patterns align with their own values and would compel centers to prioritize candidate preferences. Although the Scientific Registry of Transplant Recipients currently includes a measure of center-level offer acceptance in its program-specific reports,^28^ this measure was not designed to be easily understood by patients and is embedded in a complex 60-page document that is not patient-friendly and does not facilitate a direct comparison between transplant centers. In addition, this measure excludes all of the organ offers that bypassed a center because of prespecified center preferences that are opaque to patients and the public, further limiting its value. Third, we believe regulatory agencies must continue to reduce the emphasis on marginal differences in early posttransplant outcomes in favor of metrics that examine outcomes for all patients with ESRD. Such an approach might help shift the current focus from short-term outcomes to patient priorities, accelerating the implementation of the recent proposal of the Centers for Medicare & Medicaid Services to eliminate the use of 1-year graft and patient metrics to evaluate transplant center performance.^29^

Because any kidney can only be transplanted in a single patient, higher organ-offer acceptance rates alone will not improve the overall access to transplant. However, increased acceptance rates could lead to several positive phenomena that are associated with better outcomes and transplant rates. For example, shorter pretransplant dialysis exposure and shorter cold ischemia times improve allograft survival, which in turn reduces the number of patients returning to the waiting list for a re-transplant.^8,21,30^ Making patients aware of the organ offers that were refused on their behalf is likely to make centers more accountable to their patients. Helping centers recognize that many patients prefer having a functioning allograft with a suboptimal creatinine level over continuing to receive dialysis while waiting for a better kidney may lower the current national 20% discard rate of deceased donor kidneys.^15^ If centers were more willing to accept earlier offers for their patients, discards that result from long cold ischemia time could be avoided.^15^ Increased offer acceptance may encourage organ procurement organizations to improve deceased donor kidney recovery rates once they find that transplant centers are more willing to use these organs.

An unintended consequence of a center’s ability to decline organ offers without patient participation or awareness is that it makes an otherwise fair and objective allocation system relatively subjective.^31^ Reducing the number of times an organ is refused will potentially increase the likelihood of the organ being transplanted into the candidate for whom it was intended, according to the match run, allowing the deceased donor kidney distribution process to be consistent with the priorities incorporated into the design of the allocation system. Given previous evidence that candidates with certain characteristics, such as obesity, are disproportionately more likely to be skipped during allocation, we believe that ensuring process objectivity must be a priority.^32^

Patients can decline organ offers as well, as they often do for deceased donor kidneys of marginal quality or those labeled as having an increased risk for infectious disease transmission.^33,34^ Such refusals often occur with inadequate patient education regarding the potential risk-reward ratio. Greater transparency of organ-offer patterns and understanding of the adverse effect of extended dialysis time while waiting for a better deceased donor kidney allograft would also help candidates make better-informed choices.^8,20,21^

To our knowledge, this study is the first to analyze the implications of declining organ offers for candidates waiting for a deceased donor allograft, using the aggregated UNOS data set for all deceased donor kidneys that were eventually transplanted. The exclusion of discarded kidney offers from the UNOS data set eliminates concerns about donor organs that were not viable. However, because many discarded kidneys were potentially transplantable, this approach may underestimate the number of viable offers that candidates received.^15,16,17,18^ The greatest systemwide advantage of improving organ acceptance behavior is the reduction of organ discards and the attendant downstream consequences on encouraging organ procurement. The absence of organ-offer data before 2008 further underestimates the number of offers that prevalent wait-listed candidates received.

### Limitations

This study has several limitations. First, the center-reported reasons for declining an organ offer lack granularity. Although often the reasons to decline are multifactorial, the centers are only able to report 1 “primary” reason. This lack of detail raises concerns about the precision in the reasons. Second, we obtained only limited data after the kidney allocation policy was introduced in December 2014, when the criteria for prioritizing patients for certain organs changed. As a result, we believe additional studies are needed to identify how offer acceptance patterns have changed since the most recent policy shift.

## Conclusions

This cohort study found that kidney transplant candidates received a large number of deceased donor kidney offers that were refused on their behalf but subsequently accepted and transplanted into patients with lower priority on the match run. A death while on the waiting list was frequently preceded by multiple missed opportunities to accept an organ for transplant, which raises important questions about the current organ allocation process. Policy interventions that increase the transparency of these decisions may help maintain the objective nature of the allocation system, improve patient-centered care, and increase transplant rates in the United States.

## Notes

Supplement. **eAppendix.** Supplemental Methods **eTable 1.** Primary Reason Provided by Centers for Declining Deceased Donor Kidney Offers **eTable 2.** Secular Trends in Reasons for Deceased Donor Kidney Offer Refusal **eTable 3.** Adjusted Odds Ratio for Death on the Waitlist After Receiving at Least 1 Deceased Donor Kidney Offer, by Candidate’s State of Residence, 2008-2015 **eTable 4.** Median Number of Offers Among Those Who Died on the Waitlist Prior to Transplant, by Candidate’s State of Residence, 2008-2015 **eFigure 1.** Flow Diagram Selecting the Final Cohort and Categorizing Waitlist Outcomes of Adult Deceased Donor Kidney Transplant Candidates in the United States, 2008-2015 **eFigure 2.** Median Days Between Candidate’s First Deceased Donor Kidney Offer and Either Death on the Waitlist or Deceased Donor Kidney Transplant, by Candidate’s State of Residence, 2008-2015 **eFigure 3.** Unadjusted Odds Ratio for Death on the Waitlist After Receiving at Least 1 Deceased Donor Kidney Offer, by Candidate’s State of Residence, 2008-2015
