# Error in Author Name

**DOI:** 10.1001/jamanetworkopen.2019.14599

**Published:** 2019-10-11

In the Original Investigation titled “Association Between Declined Offers of Deceased Donor Kidney Allograft and Outcomes in Kidney Transplant Candidates,”^1^ published August 30, 2019, the surname of author Rachel E. Patzer was incorrectly spelled. This article has been corrected.^1^
