# Supplementary material for: Association Between Declined Offers of Deceased Donor Kidney Allograft and Outcomes in Kidney Transplant Candidates
Source: JAMA Netw Open. 2019 Aug 30;2(8):e1910312. doi: 10.1001/jamanetworkopen.2019.10312 (PMC6724162; doi:10.1001/jamanetworkopen.2019.10312)
Supplement: Supplement. — eAppendix. Supplemental Methods eTable 1. Primary Reason Provided by Centers for Declining Deceased Donor Kidney Offers eTable 2. Secular Trends in Reasons for Deceased Donor Kidney Offer Refusal eTable 3. Adjusted Odds Ratio for Death on the Waitlist After Receiving at Least 1 Deceased Donor Kidney Offer, by Candidate’s State of Residence, 2008-2015 eTable 4. Median Number of Offers Among Those Who Died on the Waitlist Prior to Transplant, by Candidate’s State of Residence, 2008-2015 eFigure 1. Flow Diagram Selecting the Final Cohort and Categorizing Waitlist Outcomes of Adult Deceased Donor Kidney Transplant Candidates in the United States, 2008-2015 eFigure 2. Median Days Between Candidate’s First Deceased Donor Kidney Offer and Either Death on the Waitlist or Deceased Donor Kidney Transplant, by Candidate’s State of Residence, 2008-2015 eFigure 3. Unadjusted Odds Ratio for Death on the Waitlist After Receiving at Least 1 Deceased Donor Kidney Offer, by Candidate’s State of Residence, 2008-2015 [file jamanetwopen-2-e1910312-s001.pdf]

Supplementary Online Content

Husain SA, King KL, Pastan S, et al. Association between declined offers of deceased donor kidney allograft and outcomes in kidney transplant candidates. *JAMA Netw Open*. 2019;2(8):e1910312. doi:10.1001/jamanetworkopen.2019.10312

eAppendix. Supplemental Methods

eTable 1. Primary Reason Provided by Centers for Declining Deceased Donor Kidney Offers

eTable 2. Secular Trends in Reasons for Deceased Donor Kidney Offer Refusal

eTable 3. Adjusted Odds Ratio for Death on the Waitlist After Receiving at Least 1 Deceased Donor Kidney Offer, by Candidate's State of Residence, 2008-2015

eTable 4. Median Number of Offers Among Those Who Died on the Waitlist Prior to Transplant, by Candidate's State of Residence, 2008-2015

eFigure 1. Flow Diagram Selecting the Final Cohort and Categorizing Waitlist Outcomes of Adult Deceased Donor Kidney Transplant Candidates in the United States, 2008-2015

eFigure 2. Median Days Between Candidate's First Deceased Donor Kidney Offer and Either Death on the Waitlist or Deceased Donor Kidney Transplant, by Candidate's State of Residence, 2008-2015

eFigure 3. Unadjusted Odds Ratio for Death on the Waitlist After Receiving at Least 1 Deceased Donor Kidney Offer, by Candidate's State of Residence, 2008-2015

This supplementary material has been provided by the authors to give readers additional information about their work.

©2019 Husain SA et al. *JAMA Network Open*

eAppendix. Supplemental Methods

Key Definitions:

Match Run: When a deceased donor organ is procured for transplantation, UNOS generates a list of ranked potential recipients for that organ based on matched candidate and donor characteristics and the allocation prioritization parameters (calculated panel reactive antibody sensitization, geographic boundaries, HLA DR antigen matching, time waiting for a transplant etc.). This match run data is an ordered list of all the offers to candidates until (and including) the offer when the organ was accepted for transplantation. The PTR data used only includes the match run offers for kidneys that were eventually accepted and transplanted and does not include organs that were discarded. Additionally, the match run is truncated after the accepted offer for each kidney, so all included offers are only either for the candidate who accepted the offer or for candidates who had a higher priority for the organ than the candidate who eventually accepted the offer.

Active Status: Candidates on the waitlist can be listed as status 1 (active) or status 7 (temporary inactive), and their status can be changed depending on the ability to the patient to be available and able to accept an organ offer. Those that are inactive on the waitlist do not receive any organ offers, and so all offers in this analysis are to candidates who were active on the waitlist at the time of the offer.

Variables:

Data on the candidate characteristics of interest in this study were collected using OPTN data collection forms and entered into the UNOS system by transplant center professionals at the time of the candidate's listing, with some additional updates and additional data collected at transplant, if applicable.

Age: Candidate's reported age in years at their date of listing. We calculated age at first offer by adding the difference (in years) between the candidate's first offer date and their listing date to their age in years at listing.

Sex, Ethnicity, Race: As categorized in the UNOS STAR file.

Body Mass Index (BMI): Reported continuously in kg/m^2 as calculated in the UNOS STAR file and categorized for our adjusted logistic regression model using ≥ 30 versus <30 kg/m^2 or unknown (to represent obese versus not obese candidates).

History of Diabetes/Vascular Disease: Categorized in the UNOS STAR file based on both history and duration of disease. We collapsed the categories into binary variables as any history of the disease versus no history/unknown.

Dialysis Vintage: How long a candidate has been on dialysis at their listing date and at their first offer date. Calculated as the difference (in years) between the candidate's reported dialysis start date and their listing date or first offer date. Summary statistics for dialysis duration were only calculated for candidates who were on dialysis with their dialysis start date populated; the preemptive candidates (dialysis duration of 0) were not included in the calculations.

Preemptive Status: Categorizations of whether a candidate was listed preemptively (preemptive = no dialysis vs. not preemptive/unknown) and whether their preemptive status changed: always preemptive (never on dialysis), listed preemptively but began dialysis after listing, or was already on dialysis when listed/unknown. Categories were created using the "on dialysis" variable collected by UNOS and dialysis date.

***Estimated Post Transplant Survival EPTS*:** As described by the OPTN, we calculated EPTS scores for all candidates at their time of listing and their first offer. The EPTS score is a numerical measure that ranges from 0% to 100% and is based on four factors: candidate age, if the candidate has had a prior solid organ transplant, diabetes status, and dialysis duration. Lower EPTS scores are indicative of patients expected to experience the most overall life-years (or life-benefit) while higher scores are expressive of patients estimated to experience the lowest life-benefit.

***Calculated Panel Reactive Antibodies (cPRA)*:** A measure of how sensitized a candidate is based on their most recent (end) lab Human Leukocyte Antigen testing and the national donor pool. cPRA ranges from 0% (least sensitized) to 100% (most sensitized).

Missing Data: The only variables with any missing/unknown values were BMI (1.0%), history of vascular disease (2.4%), history of diabetes (0.6%), and dialysis start date if marked as 'on dialysis' (0.2%). These candidates with missing values were combined into the respective non-obese, no vascular disease, no diabetes, and never preemptive (i.e. already on dialysis at time of listing) categories for analysis.

©2019 Husain SA et al. *JAMA Network Open*

eTable 1. Primary Reason Provided by Centers for Declining Deceased Donor Kidney Offers

Category	Reason Provided for Declining Offer	% of offer declines
Patient-Related	Patient ill, unavailable, refused, or temporarily unsuitable	2.55%
	Patient's condition improved, transplant not needed	0.01%
	Total	*2.56%*
Organ/Donor Quality	Donor age or quality	50.01%
	Not offered - minimum acceptance criteria not met	19.05%
	Organ-specific donor issue	6.66%
	Organ anatomical damage or defect	4.68%
	Organ preservation	4.61%
	Donor social history	3.77%
	Donor size/weight	3.32%
	Positive serological tests	0.51%
	Total	*92.61%*
Logistical	Distance to travel or ship	0.39%
	Operational - transplant center	0.07%
	Surgeon unavailable	0.03%
	Exceeded one hour response time	0.03%
	Heavy workload	0.02%
	Total	*0.54%*
Immunologic/ Other	Other	2.83%
	No serum	0.59%
	Positive crossmatch	0.52%
	Unacceptable antigens	0.18%
	High cPRA	0.07%
	High PRA	0.04%
	Number of HLA mismatches unacceptable	0.04%
	Total	*4.27%*

©2019 Husain SA et al. *JAMA Network Open*

eTable 2. Secular Trends in Reasons for Deceased Donor Kidney Offer Refusal

	Offer Refusal Category (%)			
**Year of Offer**	**Patient-Related**	**Organ/Donor Quality**	**Logistical**	**Immunologic/ Other**
2008	3.3	90.4	0.9	5.5
2009	2.8	90.9	0.7	5.6
2010	2.4	93.7	0.4	3.5
2011	2.6	92.4	0.5	4.5
2012	2.6	93.1	0.5	3.8
2013	2.9	92.8	0.5	3.8
2014	2.7	93.3	0.4	3.6
2015	1.7	93.6	0.5	4.3
All	2.6	92.6	0.5	4.3

©2019 Husain SA et al. *JAMA Network Open*

eTable 3. Adjusted Odds Ratio for Death on the Waitlist After Receiving At Least 1 Deceased Donor Kidney Offer, by Candidate's State of Residence, 2008-2015

Maine is used as the reference state.

State	Candidates			Deaths		Adjusted Odds Ratio	95% CI	p-value
	N	Col. %		N	Row %			
AK	319	0.11		17	5.33	1.47	(0.75, 2.86)	**0.259**
AL	6,064	2.17		1,116	18.40	5.84	(3.71, 9.21)	< 0.001
AR	1,478	0.53		84	5.68	1.61	(0.97, 2.65)	**0.064**
AZ	5,317	1.90		406	7.64	2.10	(1.32, 3.34)	0.002
CA	41,913	14.97		4,847	11.56	3.54	(2.26, 5.56)	< 0.001
CO	3,860	1.38		339	8.78	2.66	(1.67, 4.23)	< 0.001
CT	2,849	1.02		315	11.06	3.30	(2.07, 5.26)	< 0.001
DC	921	0.33		44	4.78	1.33	(0.77, 2.29)	**0.305**
DE	1,105	0.39		102	9.23	2.60	(1.58, 4.27)	< 0.001
FL	14,252	5.09		1,134	7.96	2.25	(1.43, 3.54)	< 0.001
GA	8,383	2.99		743	8.86	2.58	(1.63, 4.08)	< 0.001
HI	1,625	0.58		199	12.25	3.76	(2.34, 6.06)	< 0.001
IA	2,090	0.75		107	5.12	1.38	(0.84, 2.25)	**0.202**
ID	682	0.24		41	6.01	1.61	(0.93, 2.80)	**0.089**
IL	13,716	4.90		1,406	10.25	3.06	(1.94, 4.81)	< 0.001
IN	5,180	1.85		369	7.12	2.00	(1.26, 3.17)	0.003
KS	1,644	0.59		85	5.17	1.45	(0.88, 2.40)	**0.144**
KY	2,475	0.88		203	8.20	2.19	(1.37, 3.52)	0.001
LA	4,462	1.59		455	10.20	2.98	(1.88, 4.73)	< 0.001
MA	5,072	1.81		411	8.10	2.26	(1.43, 3.59)	0.001
MD	7,848	2.80		722	9.20	2.67	(1.69, 4.21)	< 0.001
ME	519	0.19		20	3.85	1.00	ref	ref
MI	9,569	3.42		696	7.27	1.98	(1.26, 3.13)	0.003
MN	3,787	1.35		340	8.98	2.65	(1.66, 4.21)	< 0.001
MO	4,277	1.53		303	7.08	1.91	(1.20, 3.05)	0.006
MS	3,496	1.25		368	10.53	3.05	(1.92, 4.84)	< 0.001
MT	544	0.19		50	9.19	2.54	(1.48, 4.35)	0.001
NC	8,460	3.02		690	8.16	2.32	(1.47, 3.66)	< 0.001
ND	437	0.16		35	8.01	1.99	(1.13, 3.53)	0.018
NE	1,267	0.45		59	4.66	1.22	(0.72, 2.05)	**0.456**
NH	707	0.25		48	6.79	1.82	(1.06, 3.12)	0.029
NJ	11,149	3.98		1,003	9.00	2.51	(1.60, 3.96)	< 0.001
NM	1,695	0.61		163	9.62	2.84	(1.76, 4.58)	< 0.001

©2019 Husain SA et al. *JAMA Network Open*

NV	1,758	0.63		141	8.02	2.38	(1.47, 3.86)	< 0.001
NY	20,276	7.24		2,103	10.37	2.94	(1.87, 4.62)	< 0.001
OH	8,661	3.09		728	8.41	2.33	(1.48, 3.68)	< 0.001
OK	2,463	0.88		178	7.23	2.01	(1.25, 3.23)	0.004
OR	1,434	0.51		70	4.88	1.33	(0.80, 2.22)	**0.270**
PA	13,301	4.75		1,124	8.45	2.34	(1.49, 3.69)	< 0.001
RI	641	0.23		47	7.33	2.06	(1.20, 3.53)	0.009
SC	3,781	1.35		291	7.70	2.11	(1.33, 3.37)	0.002
SD	868	0.31		60	6.91	1.74	(1.03, 2.93)	0.039
TN	5,345	1.91		425	7.95	2.19	(1.38, 3.47)	0.001
TX	26,782	9.56		2,652	9.90	2.95	(1.87, 4.63)	< 0.001
UT	1,116	0.40		56	5.02	1.36	(0.80, 2.30)	**0.252**
VA	6,813	2.43		582	8.54	2.44	(1.55, 3.87)	< 0.001
VT	320	0.11		21	6.56	1.80	(0.95, 3.40)	**0.069**
WA	3,692	1.32		188	5.09	1.44	(0.89, 2.31)	**0.135**
WI	4,113	1.47		241	5.86	1.66	(1.04, 2.65)	0.035
WV	1,212	0.43		115	9.49	2.57	(1.57, 4.19)	< 0.001
WY	303	0.11		25	8.25	2.35	(1.28, 4.34)	0.006

©2019 Husain SA et al. *JAMA Network Open*

eTable 4. Median Number of Offers Among Those Who Died on the Waitlist Prior to Transplant, by Candidate's State of Residence, 2008-2015

State	Offers before death		Days between first offer and death	
	median	IQR	median	IQR
AK	5	(3 - 8)	579	(420 - 857)
AL	30	(11 - 74)	848	(409.5 - 1432.5)
AR	13	(5 - 31.5)	412	(144.5 - 765.5)
AZ	10	(4 - 22)	618	(280 - 1085)
CA	24	(8 - 55)	757	(341 - 1300)
CO	14	(5 - 33)	707	(328 - 1170)
CT	8	(4 - 20)	712	(363 - 1130)
DC	8.5	(2.5 - 20)	421.5	(126 - 720.5)
DE	19.5	(9 - 50)	634.5	(362 - 1072)
FL	20	(7 - 45)	600.5	(283 - 992)
GA	24	(7 - 57)	673	(342 - 1101)
HI	15	(7 - 39)	785	(397 - 1217)
IA	6	(2 - 10)	368	(163 - 627)
ID	9	(2 - 15)	404	(230 - 892)
IL	12	(5 - 28)	661	(331 - 1116)
IN	12	(5 - 27)	551	(225 - 873)
KS	15	(6 - 35)	679	(310 - 1038)
KY	11	(5 - 26)	528	(214 - 956)
LA	19	(6 - 46)	573	(246 - 1026)
MA	6	(3 - 15)	625	(316 - 985)
MD	19	(6 - 44)	546	(237 - 1004)
ME	3.5	(1 - 11)	361.5	(117 - 893.5)
MI	11	(4 - 25)	567	(243.5 - 1010)
MN	7	(3 - 15)	580	(312 - 1101)
MO	12	(5 - 27)	526	(256 - 906)
MS	28.5	(8 - 73.5)	705	(297.5 - 1280)
MT	10.5	(4 - 19)	747	(441 - 1275)
NC	17	(5 - 36)	635	(314 - 1068)
ND	5	(3 - 11)	545	(171 - 1315)
NE	8	(3 - 14)	375	(138 - 783)
NH	4	(2 - 13)	589	(257 - 1145.5)
NJ	17	(6 - 45)	635	(328 - 1067)
NM	15	(5 - 30)	657	(297 - 1077)
NV	14	(5 - 32)	472	(237 - 870)
NY	17	(5 - 49)	654	(317 - 1134)
OH	18	(7 - 35)	576.5	(273 - 956.5)

©2019 Husain SA et al. *JAMA Network Open*

OK	9	(3 - 19)	536.5	(237 - 830)
OR	5	(2 - 10)	542	(281 - 1004)
PA	17	(6 - 42)	566	(255.5 - 1041)
RI	7	(2 - 15)	661	(372 - 1309)
SC	9	(3 - 26)	586	(276 - 976)
SD	9.5	(3.5 - 27)	686.5	(380 - 987)
TN	15	(5 - 39)	632	(295 - 1074)
TX	19	(6 - 51)	652	(300 - 1130.5)
UT	5	(2 - 12)	423	(191.5 - 729.5)
VA	17	(6 - 37)	703.5	(320 - 1222)
VT	6	(3 - 9)	442	(223 - 768)
WA	7	(3 - 13)	689	(293 - 1037.5)
WI	7	(3 - 17)	544	(221 - 924)
WV	9	(4 - 18)	538	(278 - 888)
WY	11	(7 - 32)	793	(449 - 1120)

©2019 Husain SA et al. *JAMA Network Open*

eFigure 1. Flow Diagram Selecting the Final Cohort and Categorizing Waitlist Outcomes of Adult Deceased Donor Kidney Transplant Candidates in the United States, 2008-2015

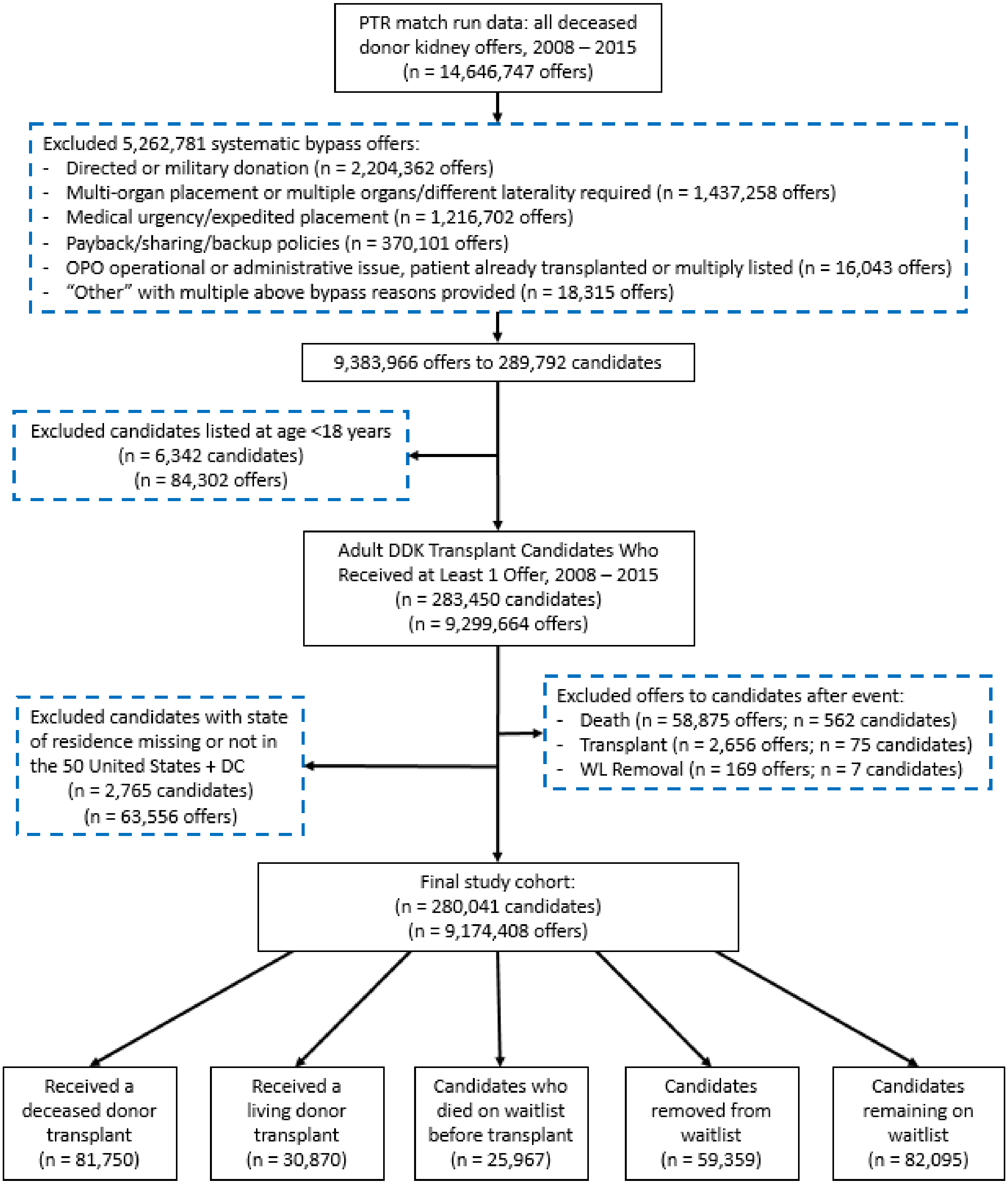

Abbreviations: PTR, potential transplant recipient; OPO, organ procurement organization; DDK, deceased donor kidney; WL, waitlist; DC, District of Columbia

©2019 Husain SA et al. *JAMA Network Open*

eFigure 2. Median Days Between Candidate's First Deceased Donor Kidney Offer and Either Death on the Waitlist or Deceased Donor Kidney Transplant, by Candidate's State of Residence, 2008-2015

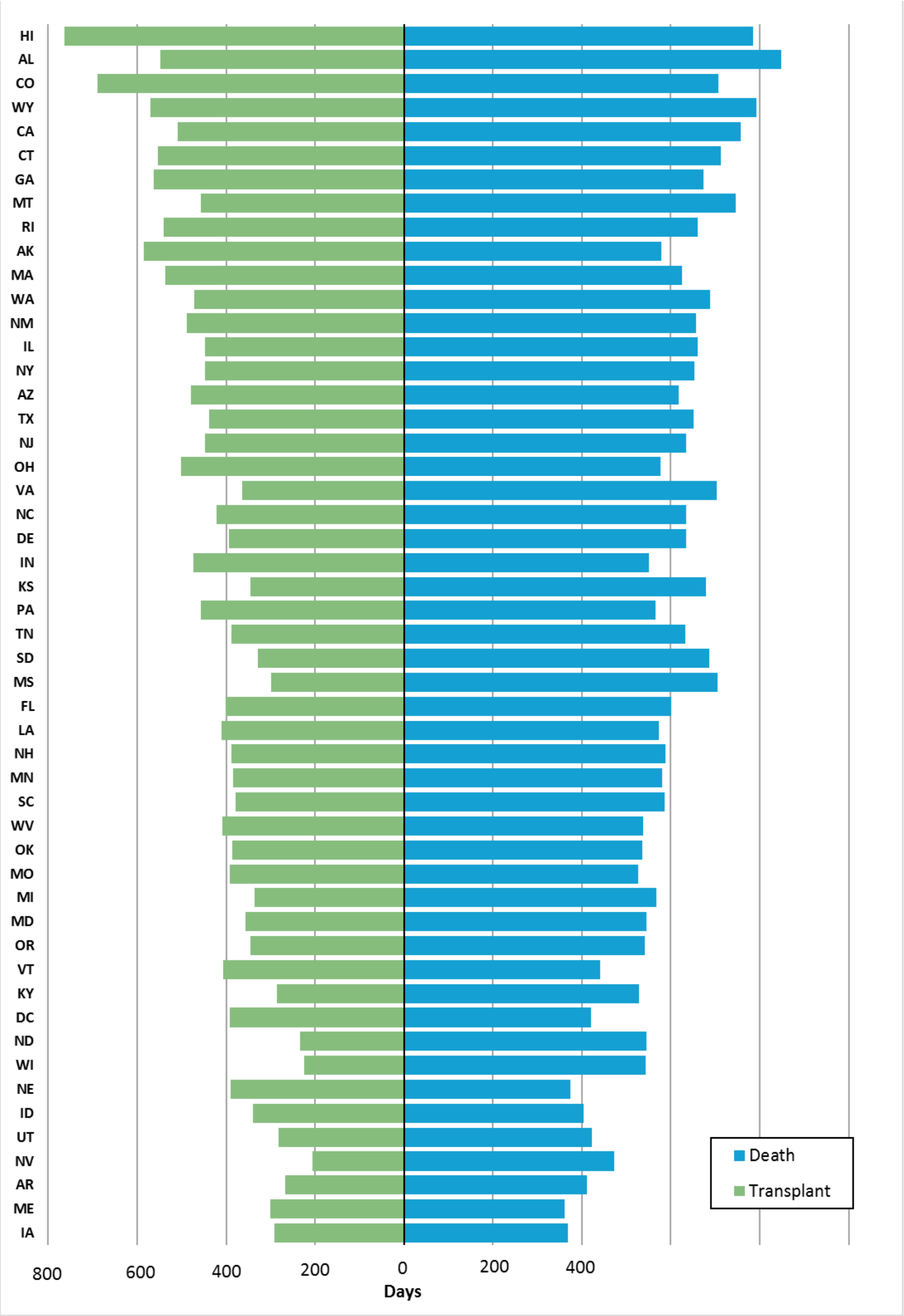

©2019 Husain SA et al. *JAMA Network Open*

eFigure 3. Unadjusted Odds Ratio for Death on the Waitlist After Receiving At Least 1 Deceased Donor Kidney Offer, by Candidate's State of Residence, 2008-2015

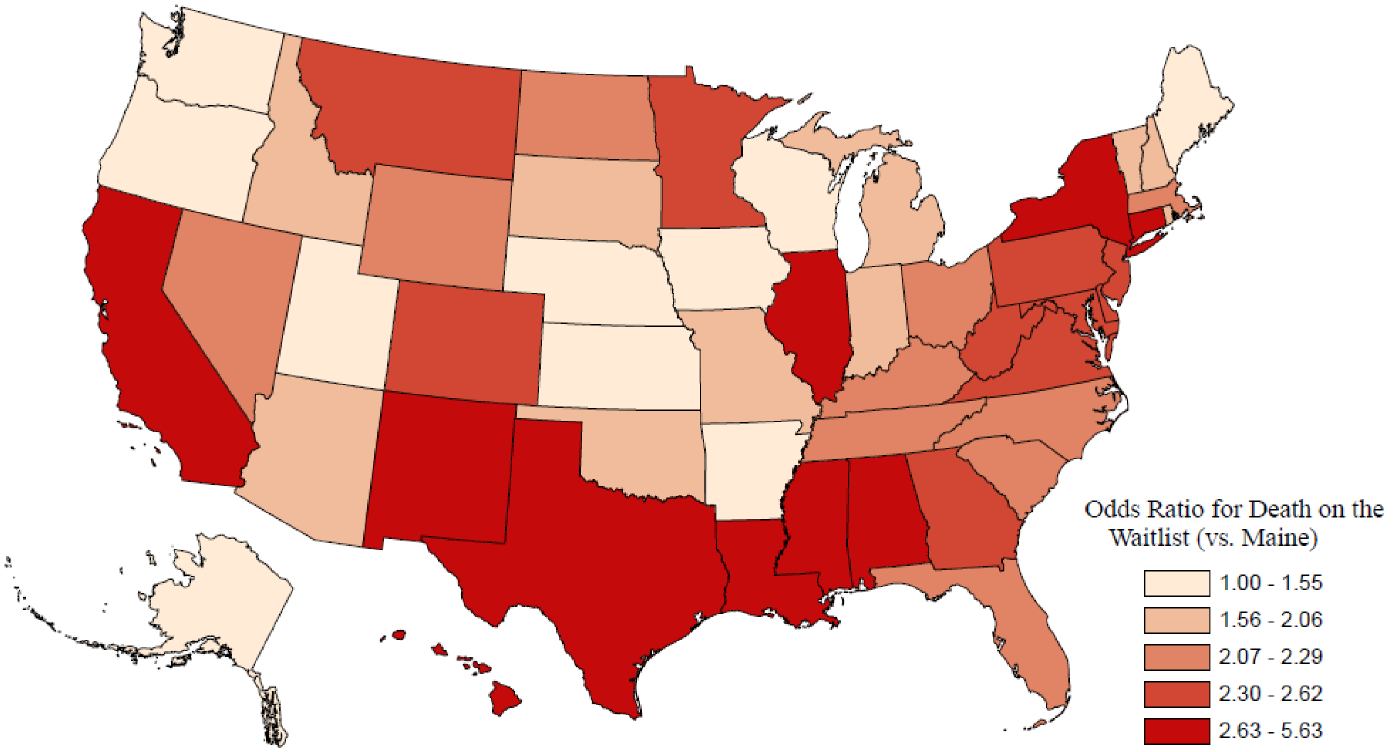

Maine (the state with the lowest proportion of deaths on the waitlist) is used as the reference state.

©2019 Husain SA et al. *JAMA Network Open*
